# Antidepressant-like activity of turmerone in behavioral despair tests in mice

**DOI:** 10.1186/1472-6882-13-299

**Published:** 2013-11-01

**Authors:** Jung-Chun Liao, Jen-Chieh Tsai, Chia-Yu Liu, Hui-Chi Huang, Lung-Yuan Wu, Wen-Huang Peng

**Affiliations:** 1 School of Pharmacy, College of Pharmacy, China Medical University, Taichung 404, Taiwan; 2 Department of Health and Nutrition Biotechnology, College of Health Science, Asia University, Taichung 404, Taiwan; 3 Department of Chinese Pharmaceutical Sciences and Chinese Medicine Resources, College of Pharmacy, China Medical University, No. 91, Hsueh-Shih Road, Taichung 404, Taiwan; 4 Department of Nursing, Jen-Teh Junior College of Medicine, Nursing and Management, No. 79-9, Sha-Luen-Hu, Xi Zhou Li, Hou-Loung Town, Miaoli County 35664, Taiwan; 5 School of Chinese Medicine for Post Baccalaureate, I-Shou university, No.1, Sec. 1, Syuecheng Rd., Dashu District, Kaohsiung City 84001, Taiwan

**Keywords:** Tumerone, Forced swimming test, Tail suspension test, Antidepressant, Monoamines

## Abstract

**Background:**

The present study was undertaken to evaluate the anti-depressive activity of turmerone after one-week administration by using a mouse forced swimming test (FST) and tail suspension test (TST).

**Methods:**

Animals were divided into four groups (n = 10 /group): control (0.9% saline), the three doses of turmerone (1.25, 2.5, 5.0 mg/kg) for one-week treatment. To assess the effect of turmerone on locomotor activity, mice were evaluated in the open-field paradigm. Forced swimming test (FST) and Tail suspension test (TST) were used to take as a measure of antidepressant activity. The probable mechanisms of action of the anti-depressive effect of turmerone was also investigated by measuring the activity of monoamine oxidase-A and corticosterone levels in the blood and the levels of monoamines in the cortex, striatum, hippocampus and hypothalamus of the mice.

**Results:**

Turmerone (2.5, 5.0 mg/kg, p.o.) significantly reduced the immobility time of mice in both the FST and TST, but it did not significantly affect the ambulatory and total movements of mice. However, hyperactivity might explain the results. In addition, turmerone decreased the corticosterone level in the blood while it increased the levels of 5-HT in cortex, striatum, hippocampus, and hypothalamus, the level of NE in striatum and hippocampus, the levels of MHPG and DOPAC in hypothalamus, the level of 5-HIAA in striatum, and the level of DA in striatum, hippocampus, and hypothalamus. Turmerone (2.5, 5.0 mg/kg) decreased the activity of MAO-A in the frontal cortex and hippocampus of mouse brain.

**Conclusions:**

After one-week administration, turmerone produced antidepressant-like effects. The mechanisms of action of anti-depressive effect of turmerone seemed to involve an increase of the monoamines level decreasing the MAO-A activity and the stress of mice.

## Background

Depression, a highly debilitating and widely distributed illness in the general population, is ranked by the World Health Organization as among the most burdensome diseases of society [1]. Affective disorder presents with depressed mood, loss of interest or pleasure, feelings of guilt or low self-worth, disturbed sleep or appetite, low energy, and poor concentration [2]. An important theory to explain the cause of depression is the monoamine hypothesis which suggests that there is impairment of monoaminergic functions and the decrease of serotonin, norepinephrine and dopamine levels [3,4]. At present, there are several types of antidepressants used in clinical practice, including tricyclic antidepressants (TCAs), selective serotonin reuptake inhibitors (SSRIs), selective reversible inhibitors of monoamine oxidase A (RIMAs), and specific serotonin–norepinephrine reuptake inhibitors (SNRIs) [5], however, these drugs can produce many side-effects, therefore, considerable efforts are being invested in the discovery of better drugs for the treatment of depression [6].

Herbal medicines such as St. John’s wort have been used as alternative therapies for depression [7]. *Curcuma longa* (Family: *Zingiberaceae*), also called *Yu-jin* in Chinese, has long been used in food and medicine. *Curcuma* drugs (e.g., *C. longa*) which were indicated for liver *qi* stagnation in Traditional Chinese Medicine, were selected for testing as a possible depression treatment [8]. Turmerone, an active constituent of *C. longa*, has been shown to possess powerful antioxidant, anti-inflammatory, anti-tumor, and anti-proliferative activities [9-13]. Inflammation is typically characterized by redness, swelling, pain, and heat. Depression is frequent in chronic pain patients and it has been suggested that pain and depression share common neurochemical mechanisms [14]. In several studies some antidepressant drugs afforded an integral alleviation of pain [15]. Curcumin has been shown to possess antidepressant-like effects in animal models commonly employed for the prediction of antidepressant activity [16-18]. It is worthy to investigate whether turmerone, an analog of curcumin [19], would possess antidepressant activity. In the present study, we aimed to test the anti-depressant effect of turmerone in mice using FST and TST. Behavioral despair tests have good predictive value for antidepressant potency in humans [20]. Moreover, we determined whether the alteration of monoamine levels and monoamine oxidase A (MAO-A) activities might predict the antidepressant properties of turmerone. We evaluated the effect of turmerone on the neuroendocrine system by measuring alterations in serum corticosterone which is involved in the mouse FST model of depression.

## Methods

### Animals

Male ICR mice (weighing around 22 g), purchased from BioLASCO Taiwan Co., Ltd. (Yi-Lan, Taiwan) were used in the present study. They were maintained at 22 ± 1°C with free access to water and food, under a 12:12 h light/dark cycle (lights on at 08:00 h). All manipulations were carried out between 9:00 and 15:00 h, with each animal used only once. All procedures in this study were performed in accordance with the NIH Guide for the Care and Use of Laboratory Animals. The experimental protocol was approved by the Committee on Animal Research, China Medical University. The minimum number of animals and duration of observations required to obtain consistent data were used.

### Chemicals and reagents

Turmerone (CAS no. 82508-15-4) was purchased from Allichem LLC (Baltimore, MD, USA). All drugs were administered by oral route. The p.o. administrations were given in a volume of 10 ml/kg body weight. Tests were performed 1 hr after administration of turmerone. The monoamine standards: norepinephrine (NE), dopamine (DA), 5-hydroxytryptamine (5-HT), 4-hydroxy-3-methoxyphenylglycol (MHPG), 3, 4-dihydroxyphenylacetic acid (DOPAC) and 5-hydroxyindole-3-acetic acid (5-HIAA) were purchased from Sigma-Aldrich (Steinheim, Germany). Ethylenediamine tetraacetic acid (EDTA) was purchased from Merck (Darmstadt, Germany). Sodium dihydrogen phosphate monohydrate (NaH_2_PO_4_ · H_2_O) was purchased from J.T. Baker (Phillipsburg, PA, USA). Sodium 1-octane sulfonate was purchased from TCI (Tokyo, Japan). Methanol and ethanol were purchased from Uni-Onward Company (Taipei, Taiwan). All other chemicals were of reagent grade or better.

### Behavior despair study

For FST and TST, animals were divided into five groups (n = 10 /group): control (0.9% saline), FLU 10 mg/kg, and the three doses of turmerone (1.25, 2.5, 5.0 mg/kg) for one-week treatment.

### Forced swimming test (FST)

The method was carried out on mice according to the method of Porsolt et al. [21]. Mice were placed in an open cylindrical container (diameter 10 cm, height 25 cm), containing 15 cm of water at 25 ± 1°C. The duration of observed immobility was recorded during the last 4 min of the 6-min testing period [22]. Immobile time was defined as the absence of active/escape directed movements (mouse floating in the water without struggling) and was scored in a blind manner by an observer [23]. Decrease in the duration of immobility during the FST was taken as a measure of antidepressant activity.

### Tail suspension test (TST)

The total duration of immobility induced by tail suspension was measured according to the method of Steru et al. [24]. Mice both acoustically and visually isolated were suspended 50 cm above the floor by adhesive tape placed approximately 1 cm from the tip of the tail. The time during which mice remained immobile was quantified during a test period of 6 min. Mice were considered immobile only when they hung passively and completely motionless.

### Open-field test

For open-field test, animals were divided into four groups (n = 10 /group): control (0.9% saline), the three doses of turmerone (1.25, 2.5, 5.0 mg/kg) for one-week treatment.

To assess the effect of turmerone on locomotor activity, mice were evaluated in the open-field paradigm (TRU SCAN Activity Monitoring Systems, Coulbourn Instruments) previously described [25]. Animals were individually placed in a box (40 × 60 × 50 cm). The mice were not habituated to the box before the test. The mice were placed in the center and their behavior was noted immediately and continued for 4 min. The parameters such as total movements, total distance, total ambulatory move time were recorded by video camera and registered in the computer. During the interval of the test the apparatus was cleaned.

Determination of monoamines and their metabolites levels in the mice frontal cortex, striatum, hippocampus, and hypothalamus.

Animals were divided into five groups (n = 10/group): control (0.9% saline), control vs. FST, FLU (10 mg/kg), and the two doses of turmerone (2.5, 5.0 mg/kg) for one-week treatment. The mice were killed after the FST which was performed 1 hr after the last administration.

Monoamines were measured according to the method of Renard et al. [26]. Briefly, mice were killed by cervical dislocation without anesthesia just after the FST. The brain was removed after a rapid dissection of frontal cortex, striatum (included the nucleus accumbens), hippocampus and hypothalamus were isolated. The four brain tissues were weighed, and placed separately in 5 ml of ice-cold homogenizing solution (8.8 mg of ascorbic acid and 122 mg of EDTA in 1000 ml of perchloric acid 0.1 M). After homogenization, the solution was centrifuged at 10,000 × g for 10 min at 4°C. Twenty microliters of the resultant supernatant was injected in the high performance liquid chromatography (HPLC) system. The levels of monoamines (NE, DA and 5-HT) and their metabolites (MHPG, DOPAC, 5-HIAA) were measured by HPLC (Waters 610) with electrochemical detection in the four brain tissues. The mobile phase [4.2 g/l citric acid monohydrate, 6.8 g/l sodium acetate trihydrate, 0.8 g/l octanesulfonic acid sodium salt, 0.05 g/l tetrasodium ethylenediamine tetraacetate, 0.02%( v/v) dibutyl amine, and 7%( v/v) methyl alcohol] was delivered at 1.0 ml/min. The reverse-phase column used was a Merk Lichrospher 100 RP-18 endcapped column with a length of 12.5 cm and an internal diameter of 4.0 mm (E. Merk 50734). The compounds were measured at +0.75 V using a Bioanalytical Systems LC-4C electrochemical detector.

### Measurements of monoamine oxidase (MAO) activity

Animals were divided into four groups (n = 10/group): control (0.9% saline), and two doses of turmerone (2.5, 5.0 mg/kg, for one week’ administration). The mice were killed after the FST which was performed 1 hr after the last administration.

Mouse brain fraction was prepared following the procedure described previously [27]. Briefly, the fraction suspended in 10 volume of cold sodium phosphate buffer (10 mM, pH 7.4, containing 320 mM sucrose), was mingled at 48°C for 20 min. The mixture was centrifuged at 15,000 g for 30 min at 8°C and the pellets were re-suspended in the same buffer. The protein concentration was adjusted to 1 mg/ml. Protein concentration was measured by the Lowry method [28] using bovine serum albumin as the standard. The MAO activity was assessed spectrophotometrically as described previously [8]. The assay mixtures contained 4 mM 5-HT as specific substrates for MAO-A, 250 ml solution of the fraction, and 100 mM sodium phosphate buffer (pH 7.4) up to a final volume of 1 ml. The reaction was allowed to proceed at 37°C for 20 min, and stopped by adding 1 M HCl (200 ml), the reaction product was extracted with 5 ml of butyl acetate (for MAO-A assay). The organic phase was measured at wavelength of 280 nm for MAO-A assay with spectrophotometer, respectively. Blank samples were prepared by adding 1 M HCl (200 ml) prior to reaction, and worked up subsequently in the same manner.

### Blood sampling and corticosterone measurement

Animals were divided into four groups (n = 6 /group): control (0.9% saline), control vs. FST, the two doses of turmerone (2.5, 5.0 mg/kg) for one-week treatment.

The animals were sacrificed 1 hr after the last administration for blood sampling between 04:00 and 06:00 p.m. The abdominal aorta was punctured under intramuscular (i.m.) ketamine hydrochloride (10%, 0.35 ml) and medetomidine hydrochloride (0.001%, 0.05 ml) anesthesia. The collected blood was heparinized and centrifuged by 400 g for 10 min. Corticosterone was measured with an automatic chemiluminescence Immunoassay system (Advia Centaur, Bayer, Bad Nauheim, Germany) [29].

### Statistical analysis

All results are expressed as mean ± SEM. Data were analyzed by one-way ANOVA followed by Scheffe’s multiple range test. The criterion for statistical significance was p<0.05. All statistical analyses were carried out by using SPSS for Windows (SPSS Inc.).

## Results

**Figure 1:**
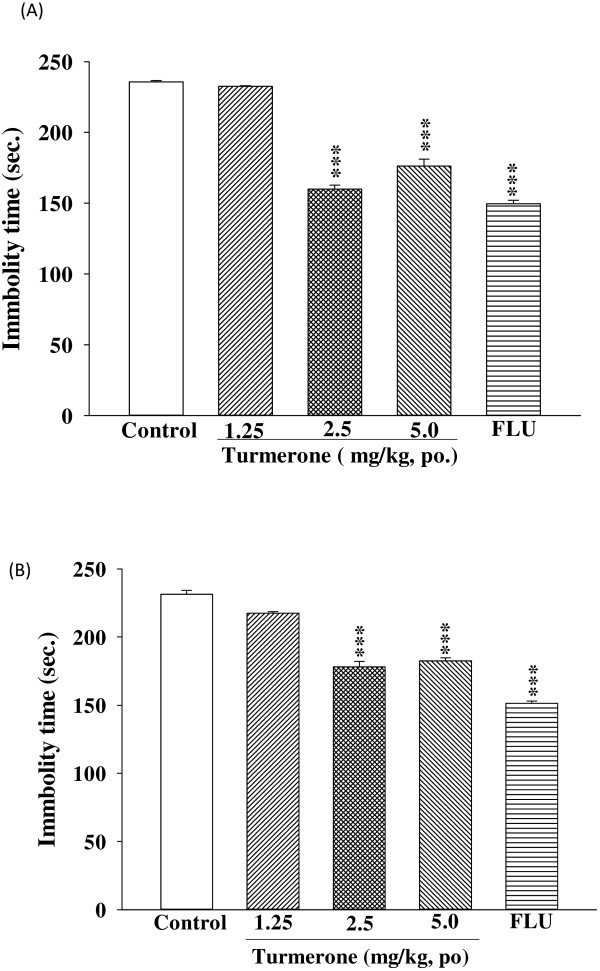
**The effect of turmerone (1.25-5.0 mg/kg, p.o.), or fluoxetine (FLU, 10 mg/kg, p.o.) on the immobility time in the forced swimming (A) and tail suspension (B) tests after one week of administration.** The values are mean ± SEM for each group (n = 10). ***p < 0.001 as compared with control group (One-way ANOVA followed by Scheffe’s multiple range test).

Turmerone caused a reduction in the immobility time in FST (F (4, 45) = 346.4) and TST (F (4, 45) = 249.8) (dose range: 1.25–5.0 mg/kg, p.o.; Figure 1).

**Figure 2:**
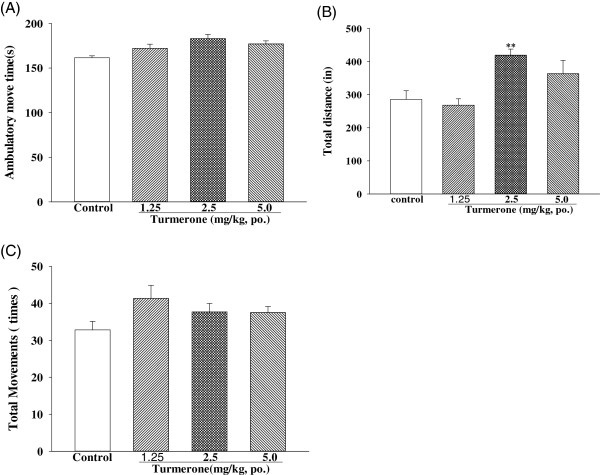
**The effects of one week of turmerone administration on: (A) total movements, (B) total distance, and (C) total ambulatory move time in the locomotion tests.** The values are mean ± SEM for each group (n = 10).

Turmerone did not affect total movement and ambulatory movement at the same doses that significantly reduced immobility response in the FST and TST (Figure 2). The total distance [F (3, 36) = 10.5] increase caused by 2.5 mg/kg turmerone may result from an increased movement speed.

As shown in Tables 1, 2, 3, and 4, turmerone (2.5 mg/kg, p.o.) increased the level of 5-HT in cortex, striatum, hippocampus, and hypothalamus, the level of NE in striatum and hippocampus, the level of MHPG and DOPAC in hypothalamus, the level of 5-HIAA in striatum, and the level of DA in striatum, hippocampus, and hypothalamus. Turmerone (5.0 mg/kg, p.o.) increased the level of 5-HT in striatum, hippocampus and hypothalamus, the level of 5-HT in cortex, the level of 5-HIAA in striatum, the level of NE in cortex and hippocampus, and the level of DA in striatum, hippocampus, and hypothalamus. Furthermore, turmerone (2.5, 5.0 mg/kg, p.o.) decreased serotonin turnover ratio (5-HIAA/5-HT) in cortex, striatum, hippocampus, and hypothalamus.

**Table 1:** Effect of turmerone on the concentrations (ng/g tissue) of monoamines and their metabolites in the cortex of mice brain

**Group**	**NE**	**MHPG**	**DA**	**DOPAC**	**5-HT**	**5-HIAA**	**5-HIAA/5-HT**
Normal	402.4 ± 19.8	243.1 ± 6.2	83.5 ± 5.6	72.2 ± 2.7	432.1 ± 22.3	127.2 ± 7.7	0.30 ± 0.03
Control + FST	200.5 ± 23.5^###^	228.9 ± 15.6	48.1 ± 2.0^##^	44.6 ± 1.5	326.9 ± 15.5^#^	255.2 ± 41.8^##^	0.75 ± 0.14^##^
Fluoxetine 10 mg/kg + FST	283.4 ± 3.3	180.4 ± 7.6	58.7 ± 1.7	47.4 ± 1.8	600.2 ± 6.1^***^	214.3 ± 3.3	0.36 ± 0.01^**^
Turmerone 2.5 mg/kg + FST	254.9 ± 9.1	172.4 ± 5.2	69.7 ± 6.3	46.9 ± 1.4	734.8 ± 18.6^***^	237.0 ± 22.0	0.33 ± 0.03^**^
Turmerone 5.0 mg/kg + FST	384.2 ± 31.3^***^	179.3 ± 5.5^*^	48.6 ± 8.6	42.5 ± 2.5	629.9 ± 25.5^***^	250.2 ± 14.0	0.42 ± 0.02

Values were the means ± SEM (n = 10). ^#^*p* < 0.05, ^# #^*p* < 0.01, ^# # #^*p* < 0.001 as compared with the normal group. ^**^p < 0.01, ^***^p < 0.001 as compared with the control + FST group. (One-way ANOVA followed by Scheffe’s test). NE: norepinephrine, MHPG: 3 methoxy-4-hydroxyphenylglycol, DA: dopamine, DOPAC: 3,4-dihydroxyphenylacetic acid, 5-HT: serotonin, 5-HIAA: *5-hydroxyindoleacetic acid.*

**Table 2:** Effect of turmerone on the concentrations (ng/g tissue) of monoamines and their metabolites in the striatum of mice brain

**Group**	**NE**	**MHPG**	**DA**	**DOPAC**	**5-HT**	**5-HIAA**	**5-HIAA/5-HT**
Normal	356.0 ± 9.6	238.0 ± 12.4	526.0 ± 9.2	157.7 ± 14.8	353.3 ± 18.7	214.1 ± 8.5	0.62 ± 0.05
Control + FST	262.3 ± 19.4	261.3 ± 9.6	342.9 ± 8.2^###^	150.1 ± 7.2	253.9 ± 9.8^#^	283.0 ± 2.9	1.07 ± 0.08^###^
Fluoxetine 10 mg/kg + FST	310.4 ± 4.7	236.2 ± 4.8	413.8 ± 5.0	144.6 ± 2.2	599.6 ± 8.8^***^	204.7 ± 3.4	0.34 ± 0.01^***^
Turmerone 2.5 mg/kg + FST	379.1 ± 43.2^*^	256.4 ± 3.3	933.5 ± 23.1^***^	129.7 ± 5.6	852.8 ± 15.3^***^	206.4 ± 5.6^*^	0.26 ± 0.01^***^
Turmerone 5.0 mg/kg + FST	327.8 ± 12.7	253.3 ± 6.8	776.9 ± 23.8^***^	137.5 ± 5.3	731.8 ± 17.6^***^	204.2 ± 20.9^*^	0.28 ± 0.03^***^

Values were the means ± SEM (n = 10). ^#^*p* < 0.05, ^###^*p* < 0.001 as compared with the normal group. ^*^p < 0.05, ^**^p < 0.01,^***^p <0.001 as compared with the control + FST group. (One-way ANOVA following by Scheffe’s test). NE: norepinephrine, MHPG: 3 methoxy-4-hydroxyphenylglycol, DA: dopamine, DOPAC: 3,4-dihydroxyphenylacetic acid, 5-HT: serotonin, 5-HIAA: *5-hydroxyindoleacetic acid.*

**Table 3:** Effect of turmerone on the concentrations (ng/g tissue) of monoamines and their metabolites in the hippocampus of mice brain

**Group**	**NE**	**MHPG**	**DA**	**DOPAC**	**5-HT**	**5-HIAA**	**5-HIAA/5-HT**
Normal	290.6 ± 23.8	244.4 ± 14.9	50.1 ± 2.0	31.9 ± 0.7	406.5 ± 14.7	198.9 ± 3.5	0.51 ± 0.03
Control + FST	220.3 ± 10.3	257.0 ± 11.8	41.9 ± 1.4	38.0 ± 1.1	216.6 ± 5.5^###^	216.1 ± 9.6	1.01 ± 0.07^###^
Fluoxetine 10 mg/kg + FST	299.7 ± 5.2	243.0 ± 5.3	49.6 ± 1.4	31.0 ± 1.8	400.0 ± 5.2^***^	194.0 ± 6.2	0.49 ± 0.02^***^
Turmerone 2.5 mg/kg + FST	398.0 ± 31.8^***^	228.0 ± 15.2	82.7 ± 2.0^***^	31.5 ± 0.9	489.1 ± 12.1^***^	176.8 ± 13.7	0.37 ± 0.03^***^
Turmerone 5.0 mg/kg + FST	386.1 ± 11.9^***^	227.0 ± 18.8	60.4 ± 1.8^***^	31.5 ± 0.8	573.1 ± 20.5^***^	185.5 ± 24.9	0.32 ± 0.05^***^

Values were the means ± SEM (n = 10). ^###^*p* < 0.001 as compared with the normal group. ^***^p <0.001 as compared with the control + FST group. (One-way ANOVA followed by Scheffe’s test). NE: norepinephrine, MHPG: 3 methoxy-4-hydroxyphenylglycol, DA: dopamine, DOPAC: 3,4-dihydroxyphenylacetic acid, 5-HT: serotonin, 5-HIAA: *5-hydroxyindoleacetic acid.*

**Table 4:** Effect of turmerone on the concentrations (ng/g tissue) of monoamines and their metabolites in the hypothalamus of mice brain

**Group**	**NE**	**MHPG**	**DA**	**DOPAC**	**5-HT**	**5-HIAA**	**5-HIAA/5-HT**
Normal	267.4 ± 7.7	201.6 ± 7.8	437.3 ± 13.0	111.0 ± 7.9	345.6 ± 21.8	145.7 ± 13.5	0.44 ± 0.06
Control + FST	207.5 ± 3.2	217.9 ± 8.8	343.6 ± 12.9^###^	183.4 ± 7.9	216.7 ± 10.4^###^	238.0 ± 13.8^##^	0.11 ± 0.05^###^
Fluoxetine 10 mg/kg + FST	224.7 ± 2.6	202.5 ± 3.1	363.1 ± 3.3	143.1 ± 3.2	422.7 ± 3.3^***^	204.6 ± 3.4	0.48 ± 0.10^***^
Turmerone 2.5 mg/kg + FST	246.1 ± 11.0	166.9 ± 16.4^*^	555.3 ± 11.4^***^	124.2 ± 7.7^*^	483.0 ± 18.5^***^	219.7 ± 14.0	0.45 ± 0.02^***^
Turmerone 5.0 mg/kg + FST	230.6 ± 13.0	196.2 ± 3.0	436.6 ± 11.6^***^	157.3 ± 16.1	539.4 ± 10.5^***^	225.4 ± 13.8	0.42 ± 0.02^***^

Values were the means ± SEM (n = 10). ^##^*p* < 0.01, ^###^*p* < 0.001 as compared with the normal group. ^*^p < 0.05, ^***^p < 0.001 as compared with the control + FST group. (One-way ANOVA followed by Scheffe’s test). NE: norepinephrine, MHPG: 3 methoxy-4-hydroxyphenylglycol, DA: dopamine, DOPAC: 3,4-dihydroxyphenylacetic acid, 5-HT: serotonin, 5-HIAA: *5-hydroxyindoleacetic acid.*

Table 5 summarizes the effect of turmerone on the activities of MAO-A in mouse brain. Turmerone (2.5, 5.0 mg/kg) decreased the activity of MAO-A in the frontal cortex and hippocampus of mouse brain.

**Table 5:** Effect of one week of turmerone administration (2.5, 5.0 mg/kg, p.o.) on MAO-A activity in different regions of the mouse brain

**Groups**	**Activity of MAO-A (U·h**^**-1**^**·mg**^**-1**^**)**			
	**Cortex**	**Striatum**	**Hippocampus**	**Hypothalamus**
Control	11.4 ± 1.1	22.6 ± 3.0	29.6 ± 1.0	40.0 ± 1.9
Turmerone 2.5 mg/kg	8.1 ±0.8^**^	22.2 ± 0.6	24.8 ± 1.2	36.6 ± 1.7
Turmerone 5.0 mg/kg	7.0 ± 0.3^**^	22.4 ± 0.6	22.7 ± 0.9^*^	37.2 ± 4.3

Values were the means ± SEM (n = 10). ^*^p < 0.05, ^**^p < 0.01 as compared with the control group. (One-way ANOVA followed by Scheffe’s test).

**Figure 3:**
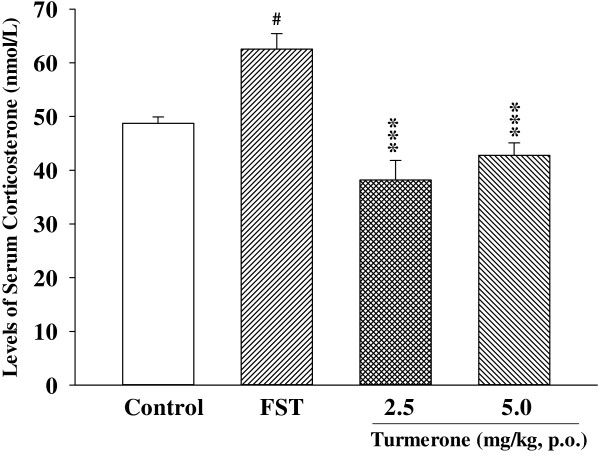
**The effects of turmerone (2.5-5.0 mg/kg) for one week’ administration on serum corticosterone levels in the mouse FST.** Data are expressed as mean ± SEM (n = 6/group). For statistical significance, ^#^*p* < 0.05 compared to vehicle-treated control group, ^***^p < 0.001 compared to vehicle-treated FST group.

As shown in Figure 3, after the one-week treatment, turmerone (2.5-5.0 mg/kg) significantly decreased the levels of serum corticosterone in mice according to the swim stress test.

## Discussion

Traditional Chinese Medicine is widely held to beneficial but generally neither the active principles nor their molecular targets are well defined [19], therefore, an understanding of the active component(s) and the mechanism(s) of action can make such medicines more acceptable.

The FST and TST are behavioral despair tests useful for probing the pathological mechanism of depression and for the evaluation of antidepressant drugs [30]. These tests are also a well-established system for screening new potent antidepressant drugs in mice [21]. Characteristic behavior scored in both tests is termed immobility, reflecting a behavioral state of despair (learned helplessness), as seen in human depression [24]. In order to investigate whether turmerone can produce chronic changes in depression-related behavior in FST and TST, we treated mice for one-week with different dosages via daily oral administration. Turmerone caused a reduction in the immobility time in FST and TST (Figure 1). The results presented here show, to our knowledge for the first time, that turmerone given orally is effective in producing significant antidepressant-like activity, when assessed in the FST and TST.

In FST and TST, anti-depressants can also be distinguished from stimulants, because stimulants cause marked motor stimulation, in contrast to antidepressants, which do not [31]. In order to determine whether turmerone actually possesses an antidepressant-like activity, we tested the locomotion counts to exclude the excitatory or inhibitory effects after administration of turmerone. Turmerone did not affect total movement and ambulatory movement at the same doses that significantly reduced immobility response in the FST and TST (Figure 2). The total distance increase caused by 2.5 mg/kg turmerone may result from an increased movement speed, so this should be further studied in the future. This hyperactivity might explain the decreasing effect of turmerone on the immobility time in FST and TST.

Most currently clinically employed antidepressants exert their effects predominantly on one monoaminergic system, although it is unlikely that pharmacological manipulation of a single neurotransmitter in relative isolation would produce changes sufficient to remedy severe neurochemical dysfunction [32]. There is abundant evidence from anatomical, electrophysiological and pharmacological studies that the interactions between neurotransmitter systems are important [31]. Four brain regions were studied: the frontal cortex, the striatum, the hippocampus, and the hypothalamus, which are involved in important behavioral functions, such as emotion, motivation and learning and memory [33,34]. Abnormal monoamine levels in the four brain regions may be relevant to the depressed state. As shown in Tables 1, 2, 3, and 4, turmerone (2.5 mg/kg, p.o.) increased the level of 5-HT in cortex, striatum, hippocampus, and hypothalamus, the level of NE in striatum and hippocampus, the level of MHPG and DOPAC in hypothalamus, the level of 5-HIAA in striatum, and the level of DA in striatum, hippocampus, and hypothalamus. Turmerone (5.0 mg/kg, p.o.) increased the level of 5-HT in striatum, hippocampus and hypothalamus, the level of 5-HT in cortex, the level of 5-HIAA in striatum, the level of NE in cortex and hippocampus, and the level of DA in striatum, hippocampus, and hypothalamus. These results indicated that the effect of turmerone on depression may be mediated via the increase in monoamines levels in the hippocampus, cortex, striatum, and hypothalamus of mice. Furthermore, turmerone (2.5, 5.0 mg/kg, p.o.) decreased serotonin turnover ratio (5-HIAA/5-HT) in cortex, striatum, hippocampus, and hypothalamus. This result indicated that turmerone increased the level of serotonin in cortex, striatum, hippocampus, and hypothalamus. MAO is an important enzyme in the metabolism of a wide range of monoamine neurotransmitters, including NE, DA, and 5-HT.

MAO-A inhibitors are efficacious for treating depression while the inhibitors of MAO-B appear to be effective in preventing and treating Parkinson’s disease. Furthermore, a positive correlation between oxidative stress and depression [35] is reported in some studies. Turmerone (2.5, 5.0 mg/kg) decreased the activity of MAO-A in the frontal cortex and hippocampus of mouse brain. The HPA axis plays a key role in the physiological response to various stressful situations [36]. Continuous activation of the HPA axis, especially abnormally increased serum corticosterone levels, leads to hormonal imbalance and even to more severe diseases such as depressive disorder both in rodents and humans [37,38]. The FST is known to be a potent activator of the HPA axis [39]. After the one-week treatment, turmerone (2.5-5.0 mg/kg) significantly decreased the levels of serum corticosterone in mice according to the swim stress test (Figure 3). The result strongly suggested that turmerone exerted antidepressant activity, at least in part, by regulating serum corticosterone levels, thus normalizing the HPA axis hyperactivity.

## Conclusion

In conclusion, turmerone reduced immobility time in the mouse FST and TST. The results suggest that the antidepressant-like effect of turmerone in FST is mediated, at least in part, by the inhibition of MAO-A, and reversed the swim stress -induced increases in serum corticosterone levels. Our study suggest that turmerone possess potent antidepressant properties.

## Competing interests

The authors declare no competing interests.

## Authors’ contributions

LYW and WHP designed the work. JCL, CYL, JCT and HCH analyzed and interpreted data for the work. All authors participated in critical manuscript revision and approved the final manuscript.

## Pre-publication history

The pre-publication history for this paper can be accessed here:

http://www.biomedcentral.com/1472-6882/13/299/prepub
